# The Incidence of Respiratory Infections and Risk Factors Among Cancer Patients Undergoing Chemotherapy at a Tertiary Care Hospital in Western Saudi Arabia

**DOI:** 10.7759/cureus.28359

**Published:** 2022-08-24

**Authors:** Abdulaziz F Alharbi, Mohammed A Bomonther, Saif AlJuaed, Suliman R Bakedo, Abdullah A Awadh, Fayssal Farahat

**Affiliations:** 1 College of Medicine, King Saud bin Abdulaziz University for Health Sciences, Jeddah, SAU; 2 Internal Medicine, King Saud bin Abdulaziz University for Health Sciences, Jeddah, SAU; 3 College of Medicine, King Saud Bin Abdulaziz University for Health Sciences, Jeddah, SAU; 4 College of Medicine/Microbiology, King Saud Bin Abdulaziz University for Health Sciences, Jeddah, SAU; 5 Infection Prevention and Control, King Abdulaziz Medical City, Riyadh, SAU; 6 College of Public Health and Health Informatics, King Saud Bin Abdulaziz University for Health Sciences, Riyadh, SAU; 7 Public Health and Community Medicine, Menoufia University, Menoufia, EGY

**Keywords:** medical icu, klebsiella pneumonia, chemotherapy, cancer, respiratory infections

## Abstract

Introduction

Cancer patients receiving chemotherapy are prone to infections because of the treatment regimens' immunosuppression.

Objectives

This study estimated the overall incidence of respiratory infections among patients undergoing chemotherapy and associated risk factors.

Methods

This study is a retrospective chart review of cancer patients at Princess Noorah Oncology Center in Western Saudi Arabia from January 2017 to December 2020.

Results

This study included 196 patients, 53.1% males and 50.5% older than 50 years. The estimated incidence of respiratory infections among participants was 8.7%, and the most commonly detected organism was *Klebsiella pneumoniae *(35.3%). The risk factors significantly associated with infection were ICU admission (p=0.001), the use of mechanical ventilation (p=0.003), and the presence of hematologic malignancy (p=0.02).

Conclusion

Future multi-center studies should employ a prospective design, including laboratory confirmation of causative organisms. Such studies may better estimate the infection-associated burden on cancer patients undergoing chemotherapy.

## Introduction

Cancer has always been a global and national health concern with increasing incidence and mortality rates. According to the WHO global cancer observatory, 19.2 million people worldwide were diagnosed with cancer in 2020, resulting in almost 10 million deaths [1]. Meanwhile, Saudi Arabia recorded 27,885 new cancer cases and 13,069 related deaths in 2020 alone [1]. Established cancer treatment plans often incorporate systemic therapy, specifically chemotherapy, as one of their modalities [2]. However, the use of chemotherapeutics contributes to patients' disease burden because of chemotherapeutics-associated adverse effects and complications [3,4]. According to a study conducted by the Christie NHS Foundation Trust, 11% of cancer patients who received systemic anti-cancer therapy died in the first 30 days of their treatment due to treatment-related complications, notably neutropenia [4]. In addition, chemotherapy suppresses the immune system, making patients prone to opportunistic infections and increasing cancer morbidity and mortality [5,6].

Respiratory infections are common among cancer patients undergoing chemotherapy [7]. Most fever cases during the onset of neutropenia are attributed to bloodstream infections and pneumonia [7,8]. Moreover, the mortality rate significantly increased among immunosuppressed cancer patients with pulmonary infiltrates (50-90%) [9]. The organisms that cause such infections include bacteria (such as *Pseudomonas aeruginosa*, *Chlamydia pneumoniae*, and *Mycobacterium avium*), fungi (such as the *aspergillus* species, the *fusarium* species, the *mucorales* species, and *Pneumocystis jiroveci*), and viruses (such as influenza viruses A and B, parainfluenza virus, cytomegalovirus, and respiratory syncytial virus) [7, 10-12]. A shift from gram-negative to gram-positive bacteria has been reported as the cause of most respiratory infections among cancer patients. At the same time, fungi were found to be the most challenging infectious organism to treat [13]. Aside from the direct damage that these infections inflict on the lungs, accompanying complications can further worsen patients' morbidity and mortality. These complications due to hematogenous dissemination include orbital and subperiosteal abscesses, meningitis, epidural abscesses, subdural empyema, brain abscesses, and toxic shock syndrome [8, 12, 13].

These chemotherapeutic complications' adverse role in patient safety and treatment success necessitates a closer examination. Accordingly, the current study estimates the incidence of respiratory infections, identifies the specific organisms that cause these infections, and pinpoints certain risk factors associated with these infections' occurrence among a population of adult patients with solid or hematological malignancies who were undergoing chemotherapy at Princess Noorah Oncology Center in King Abdulaziz Medical City, Jeddah, Western Saudi Arabia. This study focuses on the local population of Western Saudi Arabia, which the previous literature has not thoroughly examined. Additionally, it provides insights concerning the locally prevalent organisms that affect cancer patients who are receiving chemotherapy, allowing for proper, more rapid identification and treatment.

## Materials and methods

Patients

This retrospective cohort study reviewed 196 medical records of patients diagnosed with cancer and undergoing chemotherapy at Princess Noorah Oncology Center in King Abdulaziz Medical City between January 2017 and December 2020. Only adult patients (aged 18 years or older) with hematologic or solid malignancy were included, and patients who were not undergoing chemotherapy were excluded. 

Study variables

A data collection sheet was used, and data were collected from the BestCare system. The study investigators reviewed patients' medical records and collected their baseline demographic data. The incidence of laboratory-confirmed respiratory infections was recorded. Different respiratory samples (sputum, bronchoalveolar lavage, and blood) were used and diagnosed via either culture or polymerase chain reaction (PCR). The identified organism or organisms, the type of antimicrobials with which the patient was treated, and the outcome of each infection, either recovery (a negative culture or PCR), death (during the patient's hospital stay), or unknown (the patient did not report for follow up) were also recorded. In addition, the cumulative duration of each patient's chemotherapy treatment was recorded. Data pertaining to risk factors in the development of each patient's infection (diabetes mellitus, smoking, surgery, radiotherapy, ICU admission, and ventilatory support) were also recorded as present or not present. Additionally, tumor type (solid or hematological), tumor stage, and tumor histology were recorded.

Ethical consideration

This study was approved by the Institutional Review Board (IRB) of King Abdullah International Medical Research Center (KAIMRC) (IRB approval number: SP19/227/J).

Statistical analysis

Data were analyzed using IBM SPSS version 26. Descriptive statistics, including frequencies, percentages, means, medians, and SDs, were calculated. A Chi-squared or Fisher's exact test was used as appropriate to analyze categorical data. The level of significance was determined at p <0.05.

## Results

The current study reported 17 patients (8.7%) who had undergone chemotherapy and had laboratory-confirmed infections. Gram-negative bacilli accounted for most infections among this sample (n=13, 76% of the total infection cases), and the most common of these organisms was *Klebsiella pneumoniae* (n= 6, 35.3%). Fungi, particularly *Aspergillus fumigatus* and *Candida albicans*, followed (n=3, 17%). The most common tumor sites were colorectal (24.4%), the blood (17.3%), and the breast (12.7%). The occurrence of infection was significantly higher among patients with hematological malignancies (14.9%) compared to patients with solid tumors (4.9%) (p= 0.02). ICU admission (27.3% vs. 6.3%) and mechanical ventilation (37.5% vs. 7.4%) were found to significantly increase the likelihood of infection (p= 0.003). No other examined risk factor was found to be statistically associated with the occurrence of infection among the studied population (age, sex, diabetes mellitus, smoking, radiotherapy, chemotherapy duration, or surgery) (Table 1).

**Table 1:** Demographic and clinical characteristics distributed according to history of infection diagnosis. *Klebsiella pneumoniae (n=6), *Escherichia coli* (n=2), *Hemophilus influenzae* (n=2), *Aspergillus fumigatus* (n=2), *Candida albicans* (n=1), *Moraxella catarrhalis* (n=1), *Acinetobacter* (n=1), Influenza B (n=1), *Stenotrophomonas maltophilia* (n=1). ** Tumor stage was reported for solid tumors.

Characteristics	Infection* (n=17, 8.7%)	No Infection (n=179, 91.3%)	P-value
Age in years			0.19
<=50	11 (11.3)	86 (88.7)	
>50	6 (6.1)	93 (93.9)	
Gender			0.99
Male	9 (8.7)	95 (91.3)	
Female	8 (8.7)	84 (91.3)	
Diabetes Mellitus			0.58
Yes	5 (10.6)	42 (89.4)	
No	12 (8.1)	137 (91.9)	
Smoking			0.37
Yes	2 (15.4)	11 (84.6)	
No	15 (8.2)	168 (91.8)	
Tumor type			0.02
Hematological	11 (14.9)	63 (85.1)	
Solid	6 (4.9)	116 (95.1)	
Tumor stage **			0.37
I	1 (25.0)	3 (75.0)	
II	0 (0.0)	4 (100.0)	
III	1 (4.2)	23 (95.8)	
IV	4 (4.7)	81 (95.3)	
ICU admission			0.001
Yes	6 (27.3)	16 (72.7)	
No	11 (6.3)	163 (93.7)	
Ventilator			0.003
Yes	3 (37.5)	5 (62.5)	
No	14 (7.4)	174 (92.6)	
Chemotherapy duration (in months)			0.40
<5	10 (11.8)	75 (88.2)	
5-10	4 (6.3)	60 (93.8)	
>10	3 (6.4)	44 (93.6)	
Radiotherapy			0.24
Yes	1 (3.2)	30 (96.8)	
No	16 (9.7)	149 (90.3)	
Surgery			0.96
Yes	4 (8.5)	43 (91.5)	
No	13 (8.7)	136 (91.3)	

**Figure 1:**
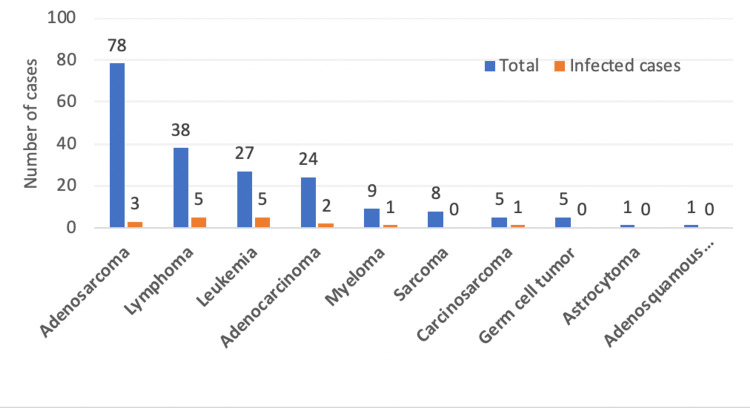
Number of diagnosed infections distributed by type of tumor.

Most infections occurred among patients with leukemia (18.5%) or lymphoma (13.2%) (Figure *1*).

**Figure 2:**
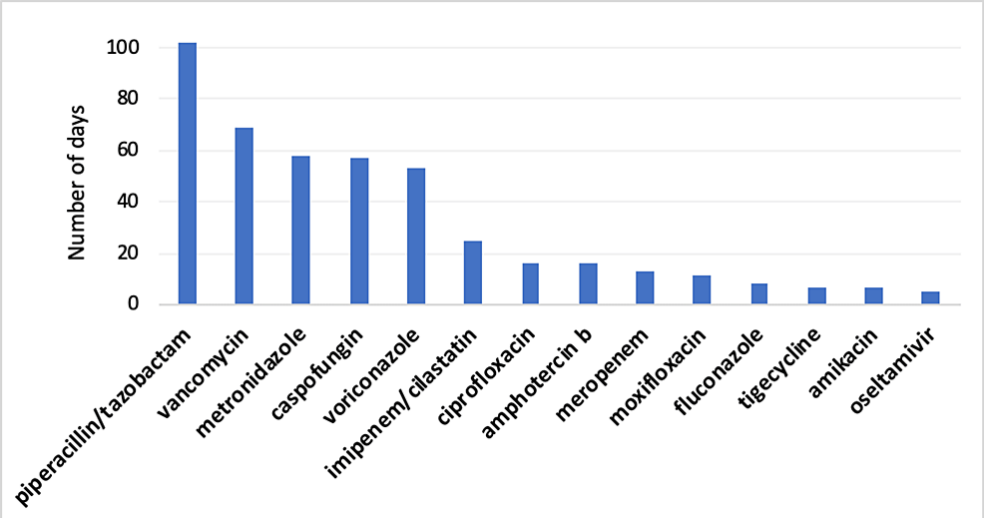
Cumulative days of antimicrobial use.

Almost half of the infected patients recovered from their infection, according to negative laboratory tests (n=8, 47.1%), while four patients died (23.5%), and the outcomes of five infected patients were unknown (29.4%). Figure 2 shows the cumulative antimicrobial treatment days for all infected patients. The average duration of antimicrobial treatment was 40 days (SD=53.75), and the median was 14 days. The minimal duration of antimicrobial treatment per patient was five days, and the maximum was 174 days. The piperacillin/tazobactam (Tazocin) antibiotic was used for the longest duration (102 days), followed by vancomycin (Vancomycin) (69 days), and oseltamivir (Tamiflu) was used for the shortest duration (five days).

## Discussion

This study aimed to estimate the occurrence of respiratory infections among patients who received chemotherapy and the associated risk factors. The overall incidence of respiratory infections in this study's sample was 8.7%, which is low compared to the corresponding 25% estimated incidence of respiratory infections among febrile cancer patients in a Chilean study [14]. The current study documented no infections of Pneumocystis jirovecii or mycobacterium species despite these organisms having accounted for a significant infectious load in previous studies [10,15,16,17]. Instead, gram-negative bacilli accounted for most infections in this population, and the most common infection-causing organism was Klebsiella pneumonia. Fujita K et al., in their study, reported unknown bacterial pneumonia as the most prevalent infection-causing (n=11, 33.3% of total infection cases), followed by Streptococcus pneumoniae (n= 3, 9.1%), and only one case of Klebsiella pneumonia (3%) [15]. The current study found the most common cancer site to be colorectal, followed by the blood and the breast, while Zheng Y et al. found the breast to be the most common cancer site (16.4%), followed by the prostate (13.6%) [18]. Moreover, the current study found the factors significantly associated with the occurrence of infections: ICU admissions, a history of mechanical ventilation, and the presence of hematological tumors. Meanwhile, Fujita K et al. found the only significant risk factor in this regard to be a history of diabetes mellitus (95% CI: 1.14-11.4, p=0.028) [15]. The duration of each patient's antimicrobial treatment in the current study ranged from five to 174 days, with a median duration of 14 days. Meanwhile, a study of patients with Hodgkin's lymphoma found the corresponding median treatment duration to be 13 days [10]. In the current study's sample, almost half of infected patients recovered from their infection, according to negative laboratory tests, and about one-quarter of infected patients (23.5%) died.

The main limitations of the current study are related to its retrospective data analysis. The outcomes of almost one-third of the cases were unknown. The study's use of laboratory findings to confirm infection also constitutes a limitation. In most cases involving febrile neutropenia, for example, the oncologist initiated broad-spectrum antibiotics empirically, based on ESMO guidelines, without laboratory confirmation of the infection­ [19]. This clinical practice has limited our ability to identify specific causative organisms. Another limitation is related to this study's small sample size, which may have affected the study's statistical power and our findings' generalizability to other institutions. 

## Conclusions

The current study reported a low incidence of laboratory-confirmed respiratory infections among cancer patients undergoing chemotherapy. Klebsiella pneumoniae was found to be the most common infection-causing organism, and the risk of infection was associated with ICU admission, mechanical ventilation, and hematological malignancy. This study's estimate of respiratory infections' true burden may have been affected by clinicians' empirical use of antibiotics and their lack of laboratory confirmation of the causative organisms.

This study highlights the importance of robust surveillance for the early detection of infection, especially among such high-risk populations as cancer patients undergoing chemotherapy. Future studies may benefit from a prospective design in which the laboratory-confirmed analysis of samples may better estimate patients' infection-associated burden.

## References

[1] Sung H, Ferlay J, Siegel RL, Laversanne M, Soerjomataram I, Jemal A, Bray F (2021). Global Cancer Statistics 2020: GLOBOCAN estimates of incidence and mortality worldwide for 36 cancers in 185 countries. CA Cancer J Clin.

[2] DeVita VT Jr, Chu E (2008). A history of cancer chemotherapy. Cancer Res.

[3] Zaorsky NG, Churilla TM, Egleston BL, Fisher SG, Ridge JA, Horwitz EM, Meyer JE (2017). Causes of death among cancer patients. Ann Oncol.

[4] Madmoli M (2018). Evaluation of chemotherapy complications in patients with cancer: a systematic review. Int J Res Stud Sci Eng Technol.

[5] Zitvogel L, Apetoh L, Ghiringhelli F, Kroemer G (2008). Immunological aspects of cancer chemotherapy. Nat Rev Immunol.

[6] Khoja L, McGurk A, O'Hara C, Chow S, Hasan J (2015). Mortality within 30 days following systemic anti-cancer therapy, a review of all cases over a 4 year period in a tertiary cancer centre. Eur J Cancer.

[7] Vento S, Cainelli F, Temesgen Z (2008). Lung infections after cancer chemotherapy. Lancet Oncol.

[8] Rolston KV, Bodey GP, Safdar A (2007). Polymicrobial infection in patients with cancer: an underappreciated and underreported entity. Clin Infect Dis.

[9] Murray PV, O'Brien ME, Padhani AR, Powles R, Cunningham D, Jeanes A, Ashley S (2001). Use of first line bronchoalveolar lavage in the immunosuppressed oncology patient. Bone Marrow Transplant.

[10] Kim T, Choi SH, Kim SH (2013). Point prevalence of Pneumocystis pneumonia in patients with non-Hodgkin lymphoma according to the number of cycles of R-CHOP chemotherapy. Ann Hematol.

[11] Arjeyni Y, Goudarzi H, Eslami G, Faghihloo E (2017). Viral respiratory infections in patients with cancer. Int J Cancer Manag.

[12] Davoudi S, Kumar VA, Jiang Y, Kupferman M, Kontoyiannis DP (2015). Invasive mould sinusitis in patients with haematological malignancies: a 10 year single-centre study. J Antimicrob Chemother.

[13] Zembower TR (2014). Epidemiology of infections in cancer patients. Cancer Treat Res.

[14] Fuentes G, Venegas C, Dreyse J, Rabagliati R, Saldias F (2014). Clinical characteristics of respiratory infections in patients with cancer. Revista Chilena De Enfermedades Respiratorias.

[15] Fujita K, Kim YH, Kanai O, Yoshida H, Mio T, Hirai T (2019). Emerging concerns of infectious diseases in lung cancer patients receiving immune checkpoint inhibitor therapy. Respir Med.

[16] Waks AG, Tolaney SM, Galar A (2015). Pneumocystis jiroveci pneumonia (PCP) in patients receiving neoadjuvant and adjuvant anthracycline-based chemotherapy for breast cancer: incidence and risk factors. Breast Cancer Res Treat.

[17] Im Y, Lee J, Kim SJ, Koh WJ, Jhun BW, Lee SH (2020). Development of tuberculosis in cancer patients receiving immune checkpoint inhibitors. Respir Med.

[18] Zheng Y, Chen Y, Yu K (2021). Fatal infections among cancer patients: a population-based study in the United States. Infect Dis Ther.

[19] Klastersky J, de Naurois J, Rolston K, Rapoport B, Maschmeyer G, Aapro M, Herrstedt J (2016). Management of febrile neutropaenia: ESMO Clinical Practice Guidelines. Ann Oncol.

